# Behavioral health outcomes and social determinants of health in children with diabetes and juvenile arthritis

**DOI:** 10.21203/rs.3.rs-3610878/v1

**Published:** 2023-11-28

**Authors:** Francesca Lupini, Tamar B Rubinstein, Eleanor R Mackey, Sangeeta Sule

**Affiliations:** University of South Carolina; Children’s Hospital at Montefiore; Children’s National Medical Center: Children’s National Hospital; Children’s National Medical Center: Children’s National Hospital

**Keywords:** juvenile rheumatology, mental health, psychosocial outcomes, school outcomes, pediatric/youth, parent outcomes, diabetes

## Abstract

**Objective:**

Children with chronic illnesses, including arthritis, are at increased risk for adverse psychosocial outcomes influenced by social determinants of health (SDOH). Comparing psychosocial outcomes in families affected by juvenile arthritis compared to other chronic illnesses may help identify areas in need of special attention vs areas that may be addressed through adopting other disease examples’ care models. We examined child and parent behavioral health outcomes for families with juvenile arthritis compared to diabetes, accounting for SDOH.

**Methods:**

Secondary data analysis of the National Survey of Children’s Health including 365 children (<18yrs) with arthritis and 571 children with diabetes. Psychosocial outcomes were depression, anxiety, ADHD, physical pain, behavioral problems, and treatment for mental health. School outcomes were school engagement, school absence, involvement in clubs/organization, and involvement in organized activities. Parent outcomes were family resilience, emotional support, coping with daily demands of raising a child, job change due to problems with childcare, and parent mental health. SDOH variables were food insecurity, food/cash assistance, unsafe neighborhood, detracting neighborhood elements, parent education, households earning <100% of the federal poverty line. Logistic regression analyses were utilized to examine variation in child and parent outcomes, variation in SDOH, and the role of SDOH.

**Results:**

Children with arthritis experienced significantly more physical pain, anxiety, depression, ADHD, and behavior problems compared to children with diabetes. Children with arthritis were more likely to see a mental health professional and get treatment for problems with emotions/behaviors. When considering SDOH, children with arthritis were still more likely to experience adverse psychosocial outcomes but were no longer more likely to get treatment. Children with arthritis had increased likelihood of school absence and were less involved in organized activities than children with diabetes. Parents of children with arthritis had poorer mental health than parents of children with diabetes. SDOH were more prevalent in children with arthritis than children with diabetes.

**Conclusions:**

Increased risk for adverse psychosocial outcomes in youth with arthritis compared to youth with diabetes indicates a need to mirror endocrinology models of care in rheumatology clinics. The role of SDOH highlights the need for regular SDOH screening in clinic.

## Introduction

Juvenile arthritis is the most common pediatric rheumatologic condition, with approximately 220,000 children and adolescents in the US affected (1). Juvenile arthritis is characterized by joint inflammation which typically results in chronic pain and functional impairment and as such, can adversely affect their quality of life (2,3).

There is a growing body of literature that indicates that living with a chronic condition, like juvenile arthritis, can negatively impact psychosocial functioning and behavioral health, particularly in children (4,5). Previous research has evidenced that youth with juvenile arthritis experience adverse psychosocial outcomes, including depressive and anxiety symptoms, internalizing and externalizing problems, and poor health-related quality of life, at a higher rate than their healthy peers (6–8). Importantly, elevated depression and anxiety in children with juvenile arthritis is associated with worse disease-related outcomes, including worse physical functioning, more tender/swollen joints, worse disability, and worse pain (6,9,10).

Chronic illnesses may also impact school outcomes, especially regarding attendance and participation (11). For instance, children living with arthritis may need to attend more doctor’s appointments and experience increased physical pain, which may impact their school attendance. School absenteeism in children with juvenile arthritis is associated with disease activity and increased pain (12–14). Qualitative research in children with juvenile arthritis and their families cite social concerns such as a desire for normalcy, physical concerns, such as a lack of accommodations, as well as stigma and misunderstanding of the disease as barriers in academic settings (15,16).

Family systems are also impacted by the responsibility of caring for a child with a chronic condition. This may manifest in increased adverse psychosocial outcomes for the child directly impacted by the condition, as well as their parents. Parents of children with juvenile arthritis experience poor psychological outcomes, such as high psychological symptoms (17), psychiatric distress (18), and general stress (19). There is evidence that poor psychological health in parents of children with juvenile arthritis may also impact child outcomes, including both child mental well-being and physical functioning (19–21). As such, family factors such as parental mental health and family conflict are important to consider in chronic illness groups.

In addition to child and family psychosocial health, social determinants of health (SDOH) may play a critical role in impacting disease outcomes, just as they play a critical role in impacting health outcomes in children without prior chronic conditions. It is important to note that SDOH refer to non-medical factors that influence health and are reflective of historical and ongoing systems of oppression that act as barriers to adherence, functioning, and access to care for individuals and family systems and consequently contribute to health inequities (22,23). Adverse SDOH impact outcomes in youth with juvenile arthritis. For instance, youth living in areas of community poverty with less access to educational opportunities for parents, experiencing low wealth, and having to rely on public insurance experienced increased functional disability (24). Much of the literature regarding disparities in juvenile arthritis has focused on race and ethnicity, which is a social construct and often difficult to distinguish from SDOH, such as socioeconomic indicators due to historical and ongoing structural racism (25).

Although there is evidence that children with arthritis and their parents experience worse psychosocial health, much of the research has been conducted either within the illness group or comparing to healthy controls. Few studies have compared children with juvenile arthritis to another chronic illness group, such as diabetes. There may be utility in comparing across chronic illness groups to identify specific areas of disease impact, which is critical for ensuring proper screening and prevention efforts to maintain quality of life in these youth. Comparing to another chronic illness group may help identify which areas could be addressed by adopting care models from other disease groups and which require more tailored attention in this population. As such, the current study aims to: 1) to compare parent and child outcomes in children with arthritis to those with diabetes, 2) to examine variation of SDOH between the two groups, and 3) to explore whether SDOH impact differences in parent and child outcomes between the two disease groups.

## Methods

We used the 2016–2019 National Survey of Children’s Health (NSCH) cross-sectional data. This survey is conducted yearly by the United States Census Bureau and sponsored by the Maternal and Child Health Bureau of the Health Resources and Services Administration and the National Center for Health Statistics at the Center for Disease Control and Prevention. Households with children < 18 years old in the United States are randomly surveyed and parents/guardians report on behalf of their children by mail or online questionnaires. The survey includes questions on demographics, physical and emotional health, factors that relate to the well-being of children, school experiences and safe neighborhoods. This analysis was exempt by the Children’s National Hospital Institutional Review Board.

### Variable Definitions

Two groups of children were identified: 1) arthritis 2) diabetes. Children were classified as having current arthritis if they had positive response to the question: “Has a doctor or other health care provider EVER told you that this child has arthritis” and “Does this child currently have the condition?” Children were classified as having current diabetes if they had positive response to the question: “Has a doctor or other health care provider EVER told you that this child has diabetes” and “Does this child currently have the condition?” Children with resolved arthritis or diabetes were excluded from the analysis, to limit the analysis to children who experience chronic arthritis rather than acute arthritis.

Age, gender, race, Hispanic ethnicity, and weight were compared between groups. Emotional and behavioral health outcomes included comorbid depression or anxiety. Depression was defined as having a positive response to both the questions: “Has a doctor or other health care provider EVER told you that this child has depression” and “Does this child currently have the condition?” with similar paired questions for anxiety. Other variables related to mental health care were defined by NSCH as “during the past 12 months” For example, “During the past 12 months, has this child received any treatment or counseling from a mental health professional?”

School outcomes were assessed in children ages 6–17 years old and included questions about school engagement: “Does this child care about doing well in school?” and missed days of school: “During the past 12 months, about how many days did this child miss school because of illness or injury?”

Variables to define differences between parent and family outcomes included coping with daily demands of raising a child, emotional help with parenthood, job change, and parent mental health. Family resiliency was defined by NSCH as a composite measure based on responses to four survey items: “When your family faces problems, how often are you likely to do each of the following?” (a) Talk together about what to do, (b) Work together to solve our problems, (c) Know we have strengths to draw on, and (d) Stay hopeful even in difficult times.” Response options to the four items were “none of the time”, “some of the time”, “most of the time”, or “all of the time”. To be positive for each individual indicator, a response of either “most of the time” or “all of the time” was required. The composite index was noted to be positive if all four individual indicators were positive.

Differences between social determinants of health were assessed by dichotomous response to questions about food insufficiency, food or cash assistance, neighborhood amenities, presence of detracting neighborhood elements, more than a high school education of an adult in the household, and households with income < 100% of the federal poverty line.

### Statistical Analyses

Statistical analysis was performed using Stata 15 (StataCorp, College Station, Texas) with weighted point estimates and variances calculated by survey procedures to account for complex sample design. Group differences were examined using Pearson chi-square tests for categorical variables and adjusted Wald tests to compare estimated means between groups. Logistic regression was used to compare arthritis vs. diabetes. Two adjusted logistic regression models were used: 1) controlling for age, gender, race, Hispanic ethnicity and 2) controlling for social determinants of health (food insufficiency, received food or cash assistance, unsafe neighborhood, presence of detracting neighborhood elements, more than high school education of adult in the household, income level < 100% of federal poverty line). P-value < 0.05 was considered statistically significant.

## Results

### Baseline Characteristics

There were 365 children with current arthritis and 571 children with current diabetes. There was no difference in age, race, ethnicity between groups. There were more children who were identified as female with arthritis compared to diabetes (63% vs. 49%, p < 0.001).

### Psychosocial Outcomes

Table 1 provides the logistic regression model examining psychosocial outcomes. Weight status and need to see mental health were similar across illness groups. However, children with arthritis were over nine times as likely.to experience physical pain than children with diabetes (OR = 9.4, [95% CI, 6.9, 12.7], *p* < .001). In terms of mental health outcomes, children with arthritis were about twice as likely to have behavior problems, anxiety, depression, and ADHD, (OR = 1.9; OR = 2.2; OR = 1.8; OR = 1.7) than children with diabetes. Lastly, children with arthritis were 1.5 times as likely to see a mental health professional (OR = 1.5, [95% CI, 1.1, 2.0], p = .007) and receive treatment for emotional or behavioral issues (OR = 1.5, [95% CI, 1.1, 2.1], p = .02). Of note, the only outcome that attenuated when accounting for SDOH was treatment for emotional or behavioral issues, such that when controlling for SDOH, children with arthritis were no longer more likely to get treatment for emotional or behavioral issues, but were still more likely to experience behavior problems, anxiety, depression, and ADHD.

### School Outcomes

School engagement was similar across illness groups, as well as child participation in clubs or organizations. However, children with arthritis were more likely to have missed more than 7 days of school (OR = 1.3, [95% CI, 1.1, 1.8], *p* = .04). and less likely to participate in one or more organized activities than children with diabetes (OR = 0.7, [95% CI, 0.5, 0.9], *p* = .01). Notably, school absenteeism attenuated when accounting for sociodemographic differences and SDOH, while participation in organized activities did not. Interestingly, child participation in clubs or organizations emerged as significant when accounting for sociodemographic variables, such that children with arthritis were less likely to participate than children with diabetes (OR = 0.7, [95% CI, 0.5, 0.9], *p* = .01) but attenuated when accounting for SDOH.

### Parent Outcomes

Parent outcomes were similar in arthritis vs diabetes families, with no significant differences in family resilience, coping with daily demands of raising a child, emotional help with parenthood, or job change due to problems with childcare. However, parents of children with arthritis were about twice as likely to exhibit poor mental health than parents of children with diabetes (OR = 1.8, [95% CI, 1.2, 2.6], *p* = .004), even when accounting for sociodemographic variables (OR = 1.7, [95% CI, 1.1, 2.6], *p* = .01) and SDOH (OR = 1.5, [95% CI, 1.0, 2.4], *p* = .05).

## Discussion

The current study analyzed data from a large national survey of families to compare child psychosocial and school outcomes, as well as parent psychosocial outcomes in youth with juvenile arthritis to youth with diabetes. It is well-established in the literature that diabetes is highly associated with increased risk for worse psychosocial outcomes (26–29). However, we found that youth with arthritis, as well as their parents, had worse psychosocial outcomes than peers with diabetes. Children with arthritis were about twice as likely to have depression, anxiety, behavior problems, and ADHD. Strikingly, youth with arthritis were nine times as likely to experience physical pain, although this is expected as pain is a direct symptom of arthritis but not of diabetes.

In terms of school outcomes, children with arthritis were similar to their peers with diabetes in school engagement and participation in clubs or organizations but had higher rates of school absenteeism and were less likely to participate in organized activities. Previous research has found that children with chronic illnesses have increased rates of school absenteeism compared to healthy peers (11), but we found that children with arthritis have increased rates of school absenteeism compared to another chronic illness group, namely diabetes. This may be related to the increased physical pain children with arthritis experience which may cause more days absent. Chronic school absenteeism is related to both short term and long term educational attainment, risky healthy behaviors, and lower income in adulthood (30–32).

We found that although parents of children with arthritis were similar to parents of children with diabetes on most outcomes, parents of children with arthritis were about twice as likely to exhibit poor mental health than parents of children with diabetes. This finding suggests that there is a need for more parental support for parents of children with arthritis. As parental mental health and social support are associated with child health outcomes (19–21), this is a group that cannot be overlooked when it comes to opportunities for additional support. Options for peer mentorship, parent support groups, acknowledgement of parent stress by providers, and referrals for care for parents may also be an important component of care for youth with arthritis. Initial work with parents of youth with type 1 diabetes indicate benefits of peer parent coaching in this population, particularly improvements in parent depressive symptoms (33). Considering increased risk in adverse mental health parental outcomes in parents of children with arthritis compared to parents of children with diabetes in the current sample, a similar model of intervention may be beneficial for parents of children with arthritis.

Importantly, the current study examined the role SDOH play in the observed differences across chronic illness groups and found that some results attenuated when accounting for SDOH, while others did not. For instance, differences between children with arthritis and diabetes in treatment for emotional or behavioral issues did attenuate when accounting for SDOH. That is, while youth with arthritis were found to be more likely to receive treatment for emotional or behavioral issues compared to youth with diabetes, this association disappeared when controlling for SDOH. This indicates that the need for mental health services in this population is particularly impacted by SDOH. This finding is consistent with previous literature that shows disparities along SDOH in mental health outcomes and access in children (34–36). Similarly, although youth with arthritis were more likely to be absent from school, this difference also attenuated when controlling for both demographics and SDOH. Children with arthritis were also less likely to participate in organized activities than children with diabetes, although this relationship was not impacted by demographic differences but was impacted by SDOH such that when accounting for SDOH, the differences disappear.

While SDOH played a role in mental health treatment, school absenteeism, and participation in organized activities, all other differences in mental health outcomes did not attenuate when accounting for SDOH. For instance, children with arthritis are at an increased likelihood to experience areas of overlap physical pain, mental health concerns including anxiety, behavioral concerns, and ADHD, as well as treatment for mental health concerns when compared to youth with diabetes. Importantly, these outcomes were not attenuated when controlling for SDOH, indicating that beyond SDOH, children with arthritis are at a greater risk for adverse mental health outcomes, including depression, anxiety, behavior problems, and ADHD. A recent study utilizing the NSCH database found important disparities related to SDOH among children with arthritis (1). Specifically, arthritis was twice as prevalent among Black youth as White youth. Furthermore, prevalence of arthritis decreased as parental education increased and prevalence rates were higher among those who experienced food insecurity than those who did not. These findings, taken together with the present study findings, highlight the importance of regularly screening for SDOH in addition to mental health concerns in order to identify youth who may need greater support. Providers should also have avenues for mental health referrals to ensure adequate care, especially for youth experiencing poverty, food insufficiency, receiving food or cash assistance, living in an unsafe neighborhood, or a neighborhood with detracting neighborhood elements, and youth with caregivers with an educational level below high school. Given the differences between illness groups and their respective care models, future research comparing care models used in endocrinology clinics and rheumatology clinics may be important to identify existing strategies that could be applied in rheumatology.

Of note, the NSCH has since changed the format of diagnosis questions, now lumping all autoimmune diseases together into one broad category. This change is particularly interesting paired with the results of the current study, as we found distinct differences in outcomes between two autoimmune illness groups, namely arthritis and diabetes. In the next iteration of the survey, we will not be able to differentiate between these two distinct illness groups and in turn, their outcomes. The analysis presented in the current study is even more important to disseminate as it is the most recent dataset from the NSCH of its kind, with the ability to compare across illness groups.

As with any study, it is important to acknowledge the limitations of the current study. The present study utilized a national database, which limited our analyses to the variables available in this database. As such, there may be additional relevant variables of interest we were unable to analyze. Furthermore, the database was cross-sectional, which limits our ability to draw conclusions about causality and directionality. The nature of the surveys relied on responses to mailings, which may have skewed the sample to those who are interested in research and trust authorities. Lastly, the surveys were only available in English and Spanish, and may have excluded a number of populations due to language barriers.

Notably, the present study had a number of strengths, including the use of a national database that allowed for larger sample sizes than typical diabetes and arthritis studies, as well as a diverse sample pulled from across the US. In addition, while most diabetes and arthritis studies are conducted within the disease group or compared to healthy controls, the present study compared two unique auto-immune illness groups, allowing for a more nuanced understanding of the outcomes. Furthermore, this comparison opens a dialogue about comparing care models such that we can apply strategies from one illness group’s care model to the other.

## Conclusion

Children with arthritis and their parents have needs that should be addressed. Diabetes clinics have a model for integrated psychosocial health assessment, while juvenile arthritis does not. It may be in our patients’ interests to consider adopting a similar model of care as diabetes, especially given the worse outcomes of children with arthritis. We also found that some outcomes, such as mental health treatment, school absenteeism, and participation in organized activities, were impacted by SDOH, while others exist beyond the impact of SDOH. This finding highlights the importance of screening for SDOH in clinics and providing extra support for patients who are burdened with SDOH. The importance of assessing and addressing SDOH, such as limited access to resources, particularly food insecurity within the clinic setting is also a critical finding of the current study. However, in addition to SDOH screening and support, it is important to develop interventions and models of care to address risk that goes beyond SDOH. Future research is needed generally in psychosocial outcomes in juvenile arthritis, in order to address the varying levels of risk found in the current study. Future research should examine the utility of prevention and intervention programs across youth, as well as potential for resource sharing across medical specialties.

## Figures and Tables

**Table 1.** Logistic regression models examining mental health outcomes between youth with juvenile arthritis and diabetes

	Simple Regression		Multiple Regression*		Multiple Regression+	
	OR (95% CI)	*p*	OR (95% CI)	p*	OR (95% CI)	*p* +
Overweight or obese, ages 10–17 years old	1.2 (0.9–1.6)	0.2	1.1 (0.8–1.6)	0.5	1.1 (0.8–1.5)	0.4
Physical pain present	9.4 (6.9–12.7)	<0.001	11.2 (7.9–15.8)	<0.001	9.3 (6.7–12.8)	<0.001
Behavior problems (ever or current)	1.9 (1.4–2.7)	<0.001	2.6 (1.7–3.8)	<0.001	1.7 (1.2–2.5)	0.007
Anxiety (ever or current)	2.2 (1.7–3)	<0.001	2.2 (1.6–3.1)	<0.001	2.1 (1.5–2.9)	<0.001
Depression (ever or current)	1.8 (1.3–2.5)	<0.001	1.7 (1.2–2.4)	0.004	1.6 (1.1–2.2)	0.01
Sees mental health professional	1.5 (1.1–2)	0.007	1.6 (1.2–2.3)	0.003	1.5 (1.1–2)	0.02
Needs to see mental health	1.4 (0.7–2.8)	0.4	1.2 (0.5–2.9)	0.7	0.9 (0.4–2.1)	0.8
Treatment for emotional or behavioral issues	1.5 (1.1–2.1)	0.02	1.5 (1.1–2.2)	0.02	1.4 (0.9–1.9)	0.08
ADHD (ever or current)	1.7 (1.2–2.4)	0.002	2.1 (1.4–3)	<0.001	1.6 (1.1–2.3)	0.02

*Note.*

* Multiple regression controlling for age, gender, race, Hispanic ethnicity;

+ Multiple regression controlling for food insufficiency, received food or cash assistance, unsafe neighborhood, presence of detracting neighborhood elements, more than high school education of adult in the household, income level < 100% of federal poverty line; OR = odds ratio; black lines under OR indicates significance, *p*<.05.

## Data Availability

The datasets analyzed during the current study are available in the from the National Survey of Children’s Health from the Health Resources and Services Administration and Maternal and Child Health Bureau (2016–2019), available from https://www.census.gov/programs-surveys/nsch/data/datasets/nsch2016.html; https://www.census.gov/programs-surveys/nsch/data/datasets/nsch2017.html; https://www.census.gov/programs-surveys/nsch/data/datasets/nsch2018.html; https://www.census.gov/programs-surveys/nsch/data/datasets/nsch2019.html.
